# The role of DNA damage and repair in decitabine-mediated apoptosis in multiple myeloma

**DOI:** 10.18632/oncotarget.1821

**Published:** 2014-03-22

**Authors:** Ken Maes, Eva De Smedt, Miguel Lemaire, Hendrik De Raeve, Eline Menu, Els Van Valckenborgh, Steve McClue, Karin Vanderkerken, Elke De Bruyne

**Affiliations:** ^1^ Department of Hematology and Immunology-Myeloma Center Brussels, Vrije Universiteit Brussel, Brussels, Belgium; ^2^ Department of Pathology, UZ Brussel, Vrije Universiteit Brussel, Brussels, Belgium; ^3^ Oncology Translational Medicine, Janssen, Pharmaceutical Companies of Johnson & Johnson, Buckinghamshire, United Kingdom

**Keywords:** Multiple myeloma, DNA methyltransferase inhibitor, histone deacetylase inhibitor, DNA damage, DNA repair

## Abstract

DNA methyltransferase inhibitors (DNMTi) and histone deacetylase inhibitors (HDACi) are under investigation for the treatment of cancer, including the plasma cell malignancy multiple myeloma (MM). Evidence exists that DNA damage and repair contribute to the cytotoxicity mediated by the DNMTi decitabine. Here, we investigated the DNA damage response (DDR) induced by decitabine in MM using 4 human MM cell lines and the murine 5T33MM model. In addition, we explored how the HDACi JNJ-26481585 affects this DDR. Decitabine induced DNA damage (gamma-H2AX foci formation), followed by a G0/G1- or G2/M-phase arrest and caspase-mediated apoptosis. JNJ-26481585 enhanced the anti-MM effect of decitabine both *in vitro* and *in vivo*. As JNJ-26481585 did not enhance decitabine-mediated gamma-H2AX foci formation, we investigated the DNA repair response towards decitabine and/or JNJ-26481585. Decitabine augmented RAD51 foci formation (marker for homologous recombination (HR)) and/or 53BP1 foci formation (marker for non-homologous end joining (NHEJ)). Interestingly, JNJ-26481585 negatively affected basal or decitabine-induced RAD51 foci formation. Finally, B02 (RAD51 inhibitor) enhanced decitabine-mediated apoptosis. Together, we report that decitabine-induced DNA damage stimulates HR and/or NHEJ. JNJ-26481585 negatively affects RAD51 foci formation, thereby providing an additional explanation for the combinatory effect between decitabine and JNJ-26481585.

## INTRODUCTION

Multiple myeloma (MM) is a plasma cell malignancy characterized by accumulation of MM cells in the bone marrow (BM) [1]. Nowadays, patients receive induction therapy with different combinations of drugs including proteasome inhibitors, immunomodulatory agents, alkylators and corticosteroids based on risk stratification. Next, if eligible, patients undergo high dose chemotherapy followed by autologous stem cell transplantion and/or consolidation or maintenance therapy [2-8]. Even though significant prolongation of overall survival is accomplished, the vast majority of patients relapses and develops non-responsive disease, demonstrating the further need for novel drugs and new therapeutic approaches.

Epigenetic modulating agents have shown considerable preclinical and clinical efficacy in hematological malignancies [9, 10]. The cytidine analog 5-aza-2'deoxycytidine or decitabine is such an epigenetic modulating agent acting as an irreversible inhibitor of DNA methyltransferases (DNMTi). Upon replication, decitabine is incorporated into DNA thereby trapping DNMT enzymes in a covalent way resulting in DNA-protein adducts [11]. The cytotoxic effects of decitabine can then be explained by two modes of action. First, trapping of DNMT enzymes leads to depletion of DNMT and the cell loses its ability to methylate DNA. The result is a genome-wide loss of methylation leading to re-activation of silenced genes, genomic instability and related anti-tumor effects. Second, the formation of DNA-protein adducts results in the activation of a DNA damage response (DDR) that can ultimately result in apoptosis.

The activation of a DDR is initiated by recognition of DNA lesions followed by cell cycle arrest and recruitment of DNA repair proteins mediating repair of the lesion. Depending on the type of lesion, different repair pathways are elicited. Double strand break repair is mediated by homologous recombination (HR) or non-homologous end joining (NHEJ). NHEJ takes place throughout all phases of the cell cycle and is considered to be error-prone. In contrast, HR takes place during S- and G2/M-phase and is dependent on the sister chromatid and considered to be more error-free. Consequently, during S- and G2/M-phase, the balance between NHEJ and HR determines which pathway will be used to repair DNA lesions [12]. Considering DNA repair in MM, it is thought that abnormal DNA repair pathways play an important role in the disease onset, progression and occurrence of resistance. This abnormal DNA repair is the result of (epigenetic) dysregulation and/or polymorphisms of genes involved in DNA repair and an accumulation of chromosomal abnormalities in MM [13]. For example, several polymorphisms in genes involved in NHEJ have been described in MM. In addition, NHEJ activity seems aberrant in MM cell lines and this influences the response towards ionizing radiation [14]. Furthermore, the activity of HR appears increased in MM [15].

Only a few studies have addressed the involvement of DNA repair in response to decitabine. In mammalian cells, HR has been implicated in tolerance towards DNA protein cross-links [16]. Recently, it was demonstrated that in Chinese hamster ovary cells, decitabine caused DNA lesions and triggered Fanconi Anemia-dependent HR. Fanconi Anemia-defective cells appeared to be more sensitive to decitabine compared to Fanconi Anemia-wild-type cells, due to the predominance of error-prone NHEJ in Fanconi Anemia-defective cells resulting into cytotoxic chromosome aberrations [17]. However, HR and NHEJ activity upon decitabine exposure have not yet been adequately addressed in MM.

The histone deacetylase inhibitors (HDACi) form another class of epigenetic modulating agents with considerable pre-clinical anti-MM activity [10]. Furthermore, HDACi showed beneficial effects in combination with conventional agents in relapsed MM patients [18, 19]. Today, several links between acetylation, HDACi and DNA repair mechanisms have been established. It is realized now that protein acetylation influences the recruitment and expression of DNA repair proteins and therefore can be used as a target to modify DNA repair pathways in response to different DNA damaging agents [20].

There has been considerable interest in combining DNMTi and HDACi to enhance the anti-tumor effects of both agents [21]. The mechanism of action involves a broad spectrum of effects that range from true epigenetic changes, chromatin- and DNA-related effects, disruption of the acetylome and micro-environmental effects [10, 22]. Previous studies on these combinations demonstrated alterations in gene expression that may correlate with an enhanced apoptotic effect and direct modulation of downstream apoptotic effectors [23-28]. Alternatively, the combinatory effects of decitabine and HDACi could be related to the DDR and modulation of the DNA repair pathways. So it would be interesting to investigate which DNA repair pathways are activated upon decitabine treatment in MM and how HDACi can affect this. Therefore, we investigated the anti-MM effects of decitabine alone and in combination with the HDACi JNJ-26481585 (JNJ-585) using human myeloma cell lines (HMCLs) and the syngeneic murine 5T33MM model and tried to unravel the underlying mechanisms with a focus on cell cycle regulation and DNA damage/repair.

## RESULTS

### Decitabine showed anti-MM effects both *in vitro* and *in vivo*

**Figure 1:**
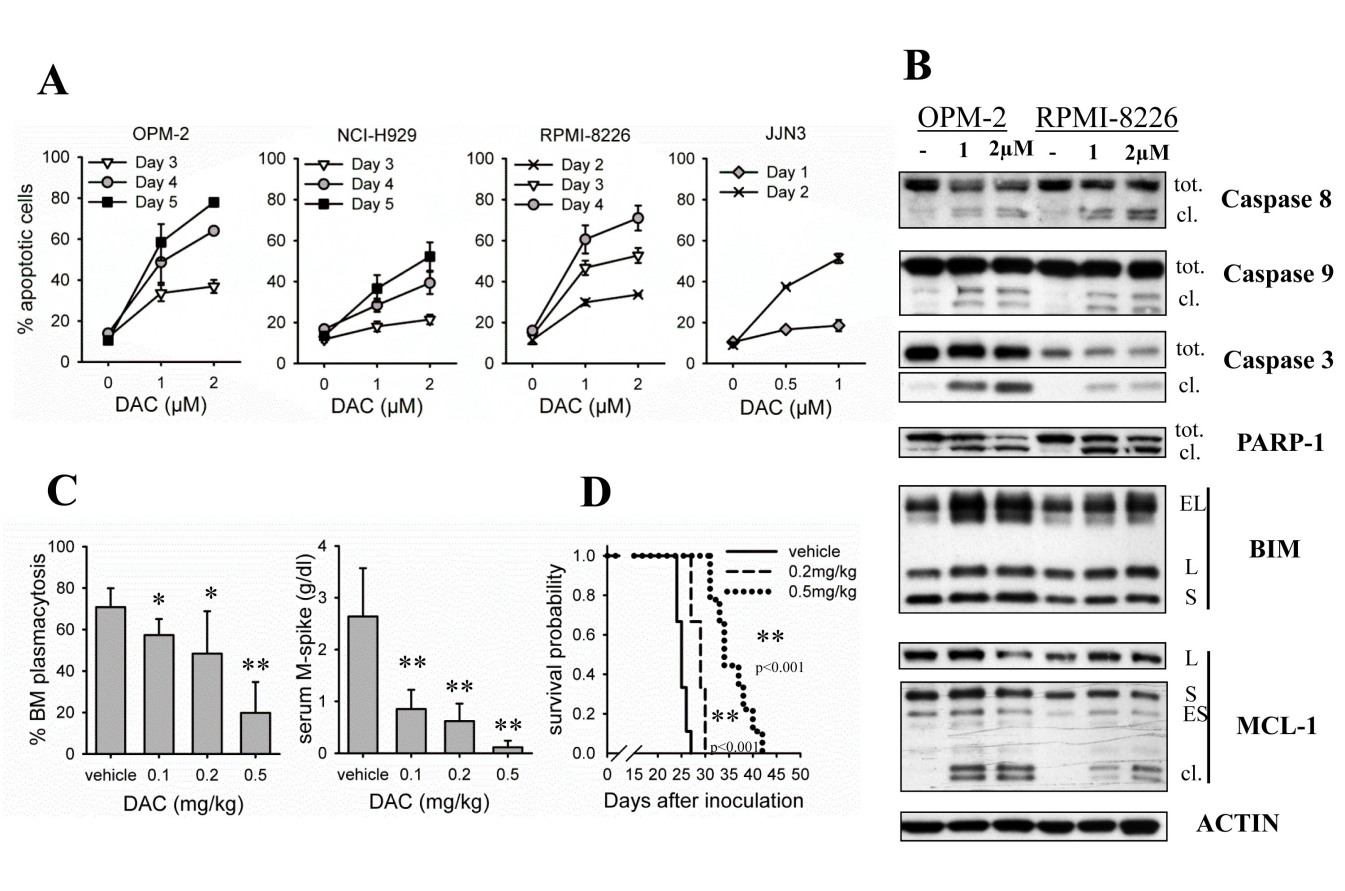
Decitabine has *in vitro*and *in vivo* anti-MM activity A: Cells were treated with different concentrations of decitabine (DAC) for indicated timepoints. Apoptosis was determined by flow cytometry using AnnexinV-FITC/7'AAD staining. Apoptotic cell percentage is the sum of annexinV+ and AnnexinV+/7'AAD+ cell percentage. Dots and error bars represent mean and SD of 3 independent experiments. B: Cells were treated for 4 days. Total protein lysates were analyzed by western blot for the presence of caspase-9, -8, -3, PARP-1, BIM and MCL-1. tot. = total; cl. = cleaved. C: C57BL/KaLwRij mice were inoculated with 5T33MM cells and treated from day 1. The experiment was terminated when the first mice showed signs of morbidity. Treatment groups were vehicle (n=5), 0.1 (n=6), 0.2 (n=5) and 0.5mpk decitabine (n=6). After sacrification, BM from hind legs was isolated. Cytospins were made and stained with May Grünwald-Giemsa. BM plasmacytosis was quantified by manual counting. Total blood was collected and the serum M-spike was measured using serum electrophoresis. * indicates p<0.05 and ** indicates p<0.001 vs. vehicle. D: Mice were treated 1 day after inoculation with 5T33MM cells. Treatment groups were vehicle (n=9), 0.2 (n=9) and 0.5mpk decitabine (n=9). Mice were sacrified individually when showing signs of morbidity. Kaplan-Meier curves were constructed and significance was evaluated by a log-rank test.

To evaluate the anti-MM effects of decitabine, we treated 4 HMCLs with different concentrations of decitabine and determined the percentage apoptotic cells. OPM-2 and NCI-H929 cells showed significant induction of apoptosis from 3 days on, while RPMI-8226 and JJN3 cells were more sensitive showing increased apoptosis already after 2 days (Figure 1A). Both in OPM-2 and RPMI-8226 cells, western blot analysis revealed a simultaneous increase in cleavage of caspase-9, -8 and -3, PARP-1 and the anti-apoptotic protein MCL-1 (Figure 1B). In addition, decitabine upregulated the pro-apoptotic protein BIM (all isoforms) in both cell lines (Figure 1B) and quantitative real-time PCR revealed that this is transcriptionally mediated (Supplementary Figure S1). To determine the potential therapeutic effects of decitabine *in vivo*, we treated 5T33MM inoculated mice with increasing doses of decitabine. Tumor load in the BM and serum M-spike were significantly lower for all decitabine treatment groups (Figure 1C). Of note, we detected no significant weight loss of the mice indicating no major toxicity (data not shown). We also performed a similar experiment in a survival setup. 5T33MM mice treated with decitabine had significant higher survival rates when compared to vehicle treated mice: 29 and 36 days for respectively 0.2mg/kg decitabine and 0.5mg/kg decitabine versus 25 days for vehicles (Figure 1D).

### Decitabine negatively affects cell cycle progression

**Figure 2:**
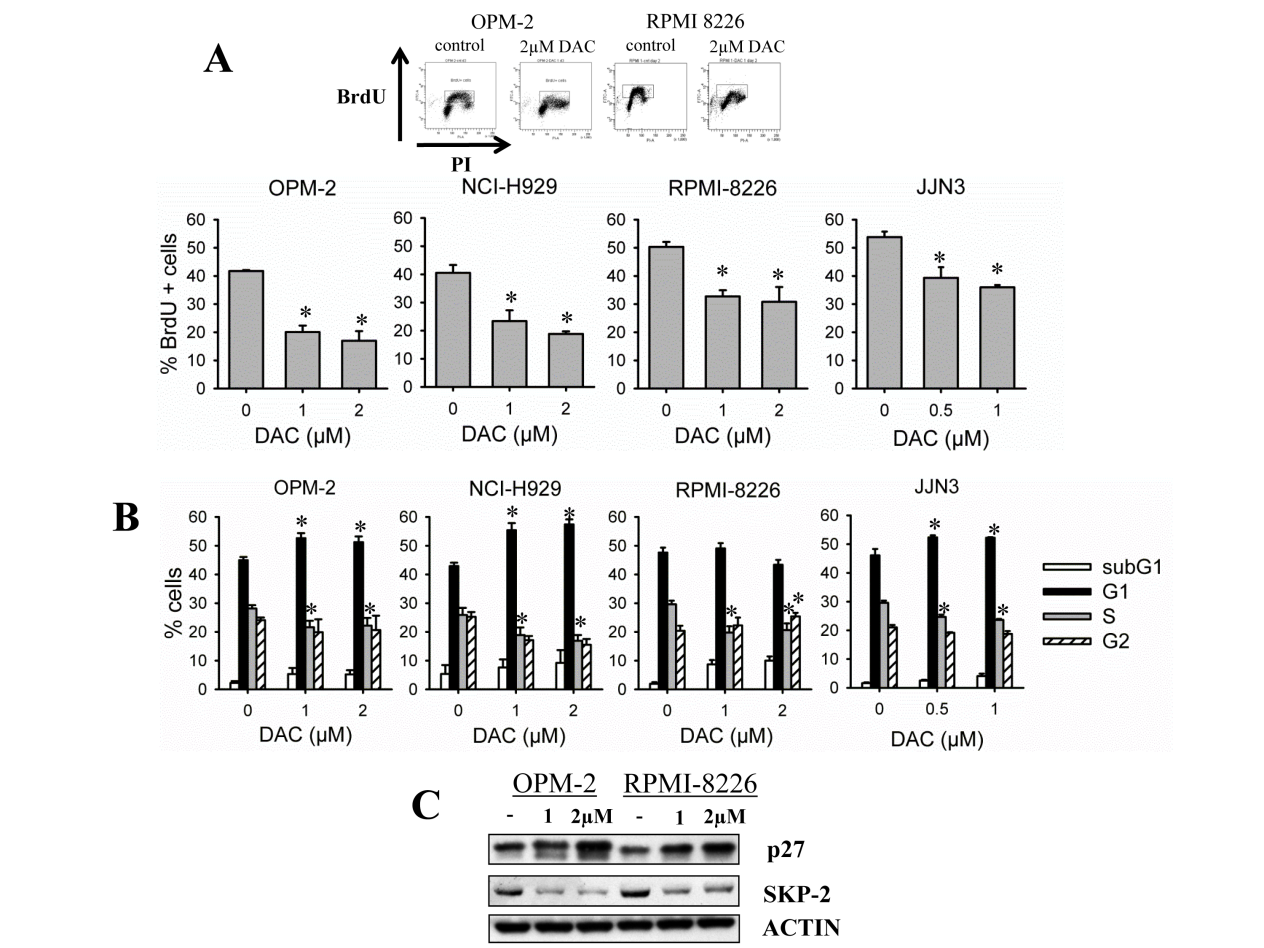
Decitabine negatively affects cell cycle progression A-B: OPM-2, NCI-H929, RPMI-8226 and JJN3 cells were treated with decitabine (DAC) for respectively 3, 3, 2 and 1 day(s). A: 2 hours prior to harvest, BrdU was added to the culture wells. Next, cells were stained with PI and anti-BrdU-FITC and analyzed by flow cytometry for DNA content and BrdU incorporation. Top: flow cytometry profiles of OPM-2 and RPMI-8226 cells. Bottom: Percentage of BrdU positive cells. B: Cell cycle profiles based on DNA content were obtained from PI histograms. Bars and error bars are mean and SD of 3 independent experiments. * indicates p<0.05 compared to control. C: Cells were treated for 3 days and total protein lysates were analyzed by western blot for the presence of p27 and SKP-2. ACTIN was used as loading control.

To investigate the mechanisms underlying the anti-MM activity of decitabine, we next determined how decitabine influences cell cycle progression in HMCLs. Therefore, we assessed DNA content together with BrdU-incorporation at timepoints when there is minimal induction of apoptosis. At control conditions, BrdU incorporation was higher for RPMI-8226 and JJN3 cells compared to OPM-2 and NCI-H929 cells. For all cell lines tested, we observed a significant decrease of BrdU incorporation upon treatment with decitabine compared to control (Figure 2A). Concordantly, DNA content analysis revealed that all cell lines showed a decrease of cells in the S-phase (Figure 2B). OPM-2, NCI-H929 and JJN3 cells accumulated all in G0/G1-phase, while RPMI-8226 cells slightly accumulated in G2/M-phase (Figure 2B). As the CDK-inhibitor p27 is known to regulate both G1 and G2 checkpoints [32], we next evaluated p27 expression and its upstream regulator SKP-2. Decitabine treatment simultaneously upregulated p27 and decreased SKP-2 expression in both OPM-2 and RPMI-8226 cells (Figure 2C).

### Decitabine induces formation of gamma-H2AX foci

**Figure 3:**
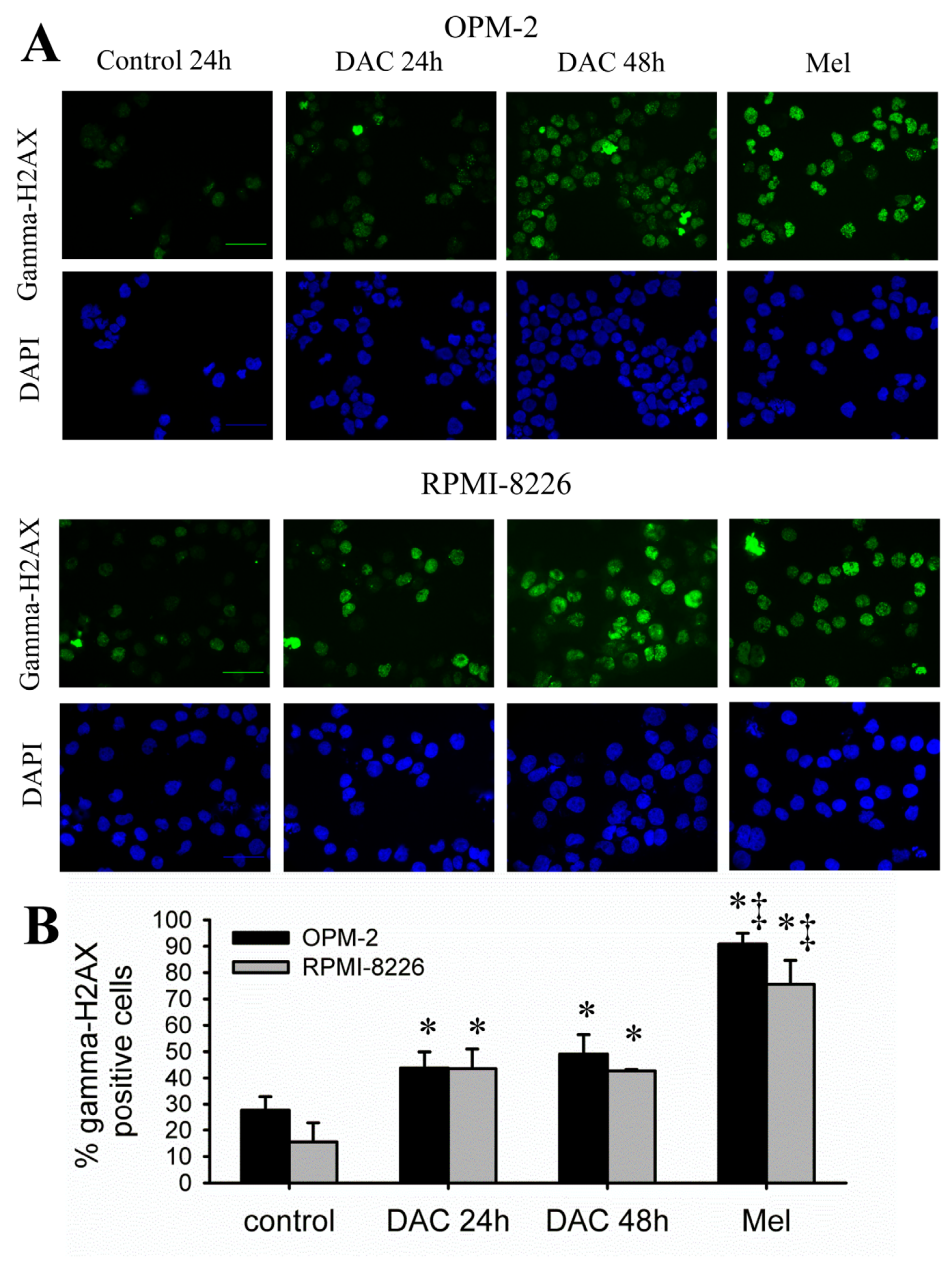
Decitabine induces gamma-H2AX foci formation A-B: Cells were treated with decitabine (DAC; 1µM) for 24 and 48 hours. Melphalan (5µM, 24 hours) was used as positive control. Next, cytospins were made and stained for gamma-H2AX. A: Immunofluorescent pictures of OPM-2 and RPMI-8226 cells after staining for gamma-H2AX and DAPI. Scale bar= 50µm. B: Quantification of the gamma-H2AX foci using ImageJ macro PZ-FociEZ. At least 100 nuclei were analyzed and nuclei with at least 10 foci were scored as positive. Data are shown as mean ± SD of 3 experiments. * indicates p<0.05 compared to basal conditions. ‡ indicates p<0.05 compared to decitabine.

The decitabine-mediated effects on cell cycle progression indicate an activation of cell cycle checkpoints in response to DNA damage. Therefore, we assessed whether decitabine could induce a DNA damage response in HMCLs. For this, we treated OPM-2 and RPMI-8226 cells with decitabine for 24 and 48 hours and analyzed gamma-H2AX foci formation, a widely used DNA damage marker. The alkylator melphalan was included as a positive control. We observed that both cell lines already have around 15 to 25% gamma-H2AX positive cells in control conditions. After decitabine treatment, the percentages of gamma-H2AX positive cells significantly increased in both OPM-2 and RPMI-8226 cells compared to control and this already after 24 hours (Figure 3A, B). Compared to melphalan, decitabine led to lower percentages of gamma-H2AX positive cells.

### The HDAC inhibitor JNJ-26481585 enhances decitabine-mediated anti-MM effects

**Figure 4:**
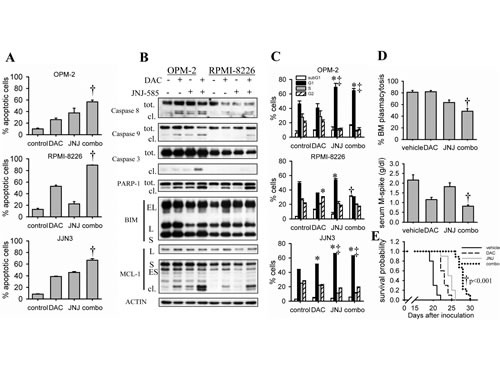
JNJ-585 enhances decitabine-mediated anti-MM effects A-C: Cells were treated with decitabine (DAC) and/or JNJ-585 for 3 days (2 days for JJN3) (A) or 2 days (1 day for JJN3) (B, C). Doses used for OPM-2 were 1µM decitabine and 2.5nM JNJ-585; for RPMI-8226 1µM decitabine and 5nM JNJ-585; for JJN3 0.5µM decitabine and 10nM JNJ-585. A: Apoptosis was determined by flow cytometry using AnnexinV-FITC/7'AAD staining. Apoptotic cell percentage is the sum of annexinV+ and AnnexinV+/7'AAD+ cell percentage. Bars and error bars are mean ± SD of 3 experiments. B: Total protein lysates were subjected to western blot analysis for the expression of caspase-9, -8, -3, PARP-1, BIM and MCL-1. ACTIN was used as loading control. C: Samples were stained with PI and cell cycle profiles based on DNA content were obtained by flow cytometry. Bars and error bars are mean ± SD of 3 experiments. D: C57BL/KaLwRij mice were inoculated with 5T33MM cells and treated from day 1. The experiment was terminated upon first signs of morbidity of the mice. Treatment groups were vehicle (n=9), 0.2mpk decitabine (n=9), 1.5mpk JNJ-585 (n=9) or the combination (n=9). After sacrification, BM from hind legs was isolated. Cytospins were stained with May Grünwald-Giemsa and BM plasmacytosis was quantified by manual counting. Total blood was collected and the serum M-spike was measured using serum electrophoresis. E: Mice were treated 1 day after inoculation with purified 5T33MM cells. Treatment groups were as follows: vehicle (n=10), 0.2mpk decitabine (n=10), 1.5mpk JNJ-585 (n=10) and the combination (n=9). Mice were sacrified individually when showing signs of morbidity. Kaplan-Meier curves were constructed and significance was evaluated by a log-rank test. * indicates p<0.05 compared to control. ‡ indicates p<0.05 compared to decitabine. † indicates p<0.05 vs. single agents.

Next, we tried to augment decitabine-mediated cell death by combining decitabine with a histone deacetylase inhibitor (HDACi), namely JNJ-26481585 (JNJ-585). Previously, we have proven that JNJ-585 has potent anti-MM effects both *in vitro* and *in vivo* [31]. To assess whether decitabine and JNJ-585 have synergistic anti-MM effects, we treated OPM-2 cells with different doses of each agent alone and in combination for 72 hours. Using the Cell Titer-Glo viability assay, we demonstrated a synergistic interaction between both agents as evidenced by combination indexes well below 1 (Supplementary Figure S2). Next, we tested whether JNJ-585 could enhance decitabine-mediated apoptosis using suboptimal doses of both agents. As shown in Figure 4, apoptosis was further enhanced in all cell lines tested and for OPM-2 and RPMI-8226 cells this was also associated with combinatory effects on cleavage of caspases, PARP-1 and MCL-1 (Figure 4A, B). Simultaneously, we also evaluated the effect of the combination on cell cycle progression. JNJ-585 alone induced a clear G0/G1-phase arrest in all cell lines, though less pronounced in RPMI-8226 cells (Figure 4C). In OPM-2 and JJN3 cells, the G0/G1-phase arrest remained after combination treatment, while in RPMI-8226 cells a clear and significant increase of cells in the subG1-phase was observed compared to single agents (Figure 4C). To confirm the *in vitro* data, we then tested if JNJ-585 could potentiate decitabine-mediated effects in the 5T33MM model. Indeed, the combination of a suboptimal dose of decitabine and JNJ-585 significantly augmented the effects of decitabine on tumor progression and survival (Figure 4D, E).

### JNJ-585 alters the DNA repair response towards decitabine-induced DNA damage

**Figure 5:**
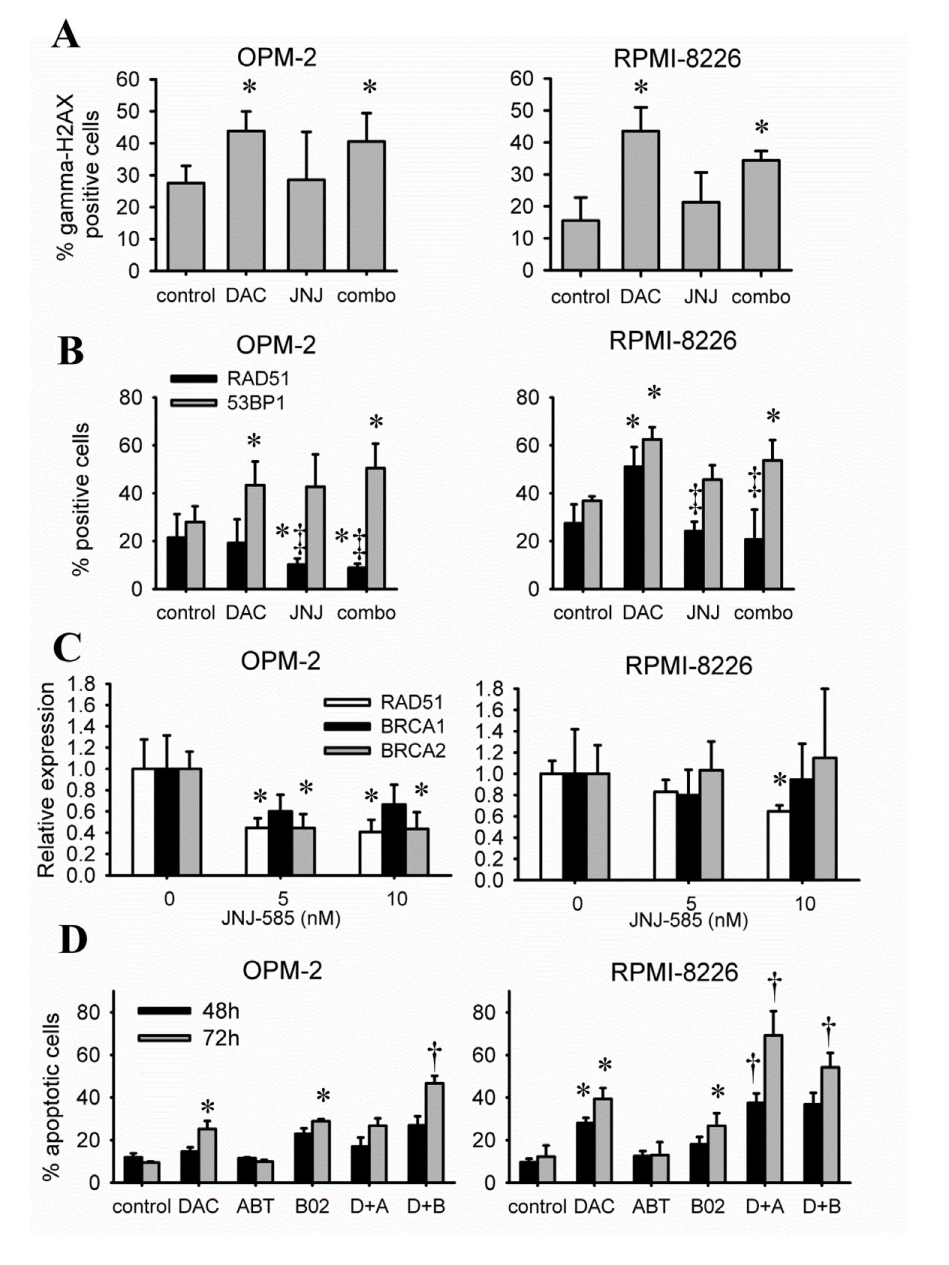
JNJ-585 affects the repair response elicited by decitabine A-B: Cells were treated with decitabine (DAC) and/or JNJ-585 for 1 day. Doses for OPM-2 were 1µM decitabine and 2.5nM JNJ-585; for RPMI-8226 1µM decitabine and 5nM JNJ-585. Next, cytospins were made and stained for gamma-H2AX (A), RAD51 and 53BP1 (B). Images were quantified using ImageJ PZFociEZ plugin. A: Quantification of gamma-H2AX foci. B: Quantification of RAD51 and 53BP1 foci. At least 100 nuclei were analyzed and nuclei with at least 10 foci were scored as positive. C: Cells were treated with JNJ-585 for 1 day. Samples were processed and used for qRT-PCR to analyze expression of RAD51, BRCA1 and BRCA2. ABL-1 was used as housekeeping gene. D: Cells were treated with decitabine (1µM) and/or B02 (10µM) or ABT-888 (10µM) for 2 or 3 days. Next, apoptosis was determined by flow cytometry using AnnexinV-FITC/7'AAD staining. Apoptotic cell percentage is the sum of annexinV+ and AnnexinV+/7'AAD+ cell percentage. Data is shown as mean ± SD of 3 experiments. * indicates p<0.05 compared to untreated conditions. ‡ indicates p<0.05 compared to decitabine. † indicates p<0.05 compared to single agents.

We next investigated the effects of JNJ-585 on decitabine-mediated DNA damage. Using immunofluorescence analysis, we found that suboptimal doses of JNJ-585 did not change the baseline percentage of gamma-H2AX positive cells in OPM-2 and RPMI-8226 cells (Figure 5A). In addition, the combination showed similar but not additional numbers of gamma-H2AX positive cells compared to decitabine (Figure 5A). This suggests that decitabine-induced DNA damage is not enhanced by JNJ-585. Next, we hypothesized that JNJ-585 might alter the DNA repair response towards decitabine, thereby enhancing the cytotoxicity of the DNA damage induced by decitabine. As previously mentioned, double strand break repair is involved in decitabine-mediated DNA lesions [16, 17]. Therefore, we analyzed the contribution of HR and NHEJ by evaluating the presence of respectively RAD51 and 53BP1 foci. Both OPM-2 and RPMI-8226 cells already showed RAD51 and 53BP1 foci formation in basal conditions (Figure 5B and Supplementary Figure S3-S6). Decitabine alone significantly increased the number of 53BP1 positive cells in both OPM-2 and RPMI-8226 cells, while RAD51 positive cells were increased only in RPMI-8226 cells (Figure 5B and Supplementary Figure S3-S6). Interestingly, JNJ-585 alone did not alter the percentage of RAD51 positive cells compared to control conditions in RPMI-8226 cells. However, the increase in RAD51 positivity by decitabine was completely abrogated by JNJ-585 (Figure 5B and Supplementary Figure S3-S6). Moreover, in OPM-2 cells, JNJ-585 even decreased the baseline presence of RAD51 foci and this was even more pronounced in the combination. Although significance was not reached, JNJ-585 slightly increased the formation of 53BP1 foci in both cell lines (Figure 5B and Supplementary Figure S3-S6). The combination increased 53BP1 foci formation to a similar extent as decitabine. We next evaluated whether JNJ-585 could modulate expression of the HR-related genes RAD51, BRCA1 and BRCA2. Quantitative real-time PCR revealed that JNJ-585 could decrease the expression of RAD51 in both RPMI-8226 and OPM-2 cells. In addition, BRCA2 expression was downregulated in OPM-2 cells. There was also tendency for downregulation of BRCA1 expression in OPM-2 cells but significance was not reached (Figure 5C).

### Targeting DNA repair pathways appears useful in combination with decitabine

The above experiments indicate that by targeting DNA repair responses, the cytotoxic effects of decitabine might be enhanced. Here, we chose to target RAD51 and PARP1/2 by respectively B02 and ABT-888. B02 is a specific RAD51 inhibitor that has not been used in MM or related disorders [33, 34], while ABT-888 (Veliparib) is a PARP1/2 inhibitor [35]. B02 alone was cytotoxic in both OPM-2 and RPMI-8226 cells as evidenced by an increased percentage of apoptotic cells while ABT-888 alone did not show any cytotoxicity (Figure 5D). Co-treatment of cells with B02 sensitized both OPM-2 and RPMI-8226 cells to decitabine as evidenced by significantly more apoptosis compared to single agents (Figure 5D). Co-treatment with ABT-888 also sensitized RPMI-8226 cells, but not OPM-2 cells, to decitabine (Figure 5D).

## DISCUSSION

Epigenetic agents and in particular DNMTi and HDACi are under intense investigation for their use in cancer therapy [21]. Better understanding of the underlying molecular effects of these agents will facilitate the translation into the clinic. Here, we investigated the possible induction of a DDR by decitabine in MM and how this is affected by JNJ-585 and different DNA repair inhibitors.

Using a panel of HMCLs, we observed the induction of apoptosis in response to decitabine at different timepoints. In addition to the HMCLs, we also demonstrate significant anti-MM effects of decitabine using the 5T33MM model. Previous work already demonstrated a difference in sensitivity to decitabine using a broad panel of HMCLs [36]. We confirm that RPMI-8226 and JJN3 cells display a rapid onset of apoptosis and thus a high sensitivity to decitabine. The effects on downstream apoptotic proteins were similar in OPM-2 and RPMI-8226 cells. Both cell lines displayed clear cleavage of caspase proteins and PARP-1 when significant amounts of apoptosis were detected. Simultaneously, pro-apoptotic BIM expression was elevated both at the transcriptional and translational level. This is in agreement with our previous study where we showed the epigenetic regulation of BIM in MM [29]. We also observed cleavage of anti-apoptotic MCL-1 simultaneously with caspase cleavage. In human, three splice variants of MCL-1 have been described, namely the anti-apoptotic isoform MCL-1L and the pro-apoptotic isoforms MCL-1S and MCL-1ES. At the post-translational level, caspase-mediated MCL-1 cleavage has also been observed [37, 38]. This supports our results that MCL-1 cleavage is caspase-mediated. It is also in line with the observation of cleavage of MCL-1 upon AZA treatment in MM1.S cells [39].

One aspect that may determine sensitivity to decitabine is the rate of decitabine incorporation. BrdU incorporation can be used to evaluate import and incorporation of nucleosides necessary for DNA replication and DNA repair. We observed that RPMI-8226 and JJN3 cells incorporate more BrdU at basal conditions compared to OPM-2 and NCI-H929 cells. In analogy, decitabine incorporation will be higher in RPMI-8226 and JJN3 cells what may result in a faster accumulation of lethal levels of DNA damage compared to OPM-2 and NCI-H929 cells. Preceding apoptosis, a significant amount of cells accumulated in the G0/G1- or G2/M-phase upon decitabine treatment. This confirms earlier work showing the anti-proliferative effects of decitabine [40, 41]. In addition, BrdU incorporation decreased upon decitabine exposure what correlates with the drop of cells in S-phase and the observed arrest. Cell cycle arrest by decitabine is often associated with p21 upregulation [40, 42]. However, the role of the CDK-inhibitor p27 herein is less clear. In association with the cell cycle arrest, we observed an upregulation of p27. It has been described that p27 protein expression is stabilized in response to DNA damage and may be part of a DDR pathway related to the detrimental effects of continuous DNA damage [43]. Furthermore, we showed a simultaneous drop in SKP-2 expression. SKP-2 is responsible for p27 degradation [44], thereby explaining the observed accumulation of p27. This indicates that SKP-2 may be downregulated in response to decitabine-mediated DNA damage what results in p27 accumulation and subsequent cell cycle arrest. Indeed, earlier reports demonstrated SKP-2 downregulation and p27 accumulation in response to DNA damage [45, 46].

Our observation that decitabine increased the percentages of cells having more than 10 gamma-H2AX foci is in agreement with previous studies showing the DNA-damaging effects of DNMTi [17, 39, 40, 47]. Compared to melphalan, decitabine led to lower percentages of gamma-H2AX positive cells demonstrating that melphalan is more potent to induce DNA damage. Also at the basal level, we could already detect a fair amount of gamma-H2AX positive cells what is in line with a previous report showing constitutive DNA damage in MM [48]. Interestingly, the induction of gamma-H2AX positive cells by decitabine was similar between RPMI-8226 and OPM-2 cells and therefore cannot explain the earlier apoptosis induction in RPMI-8226 cells. This suggest that, not only the amount of gamma-H2AX positive cells per se, but also the ability to repair decitabine-induced DNA lesions determines if a cell will undergo apoptosis. HR has been implicated in the response towards DNA-protein cross links [16]. Furthermore, Orta *et al.* showed earlier that decitabine could induce both RAD51 and 53BP1 foci formation and proposed that HR governs protection while NHEJ results in accumulation of cytotoxic chromosome aberrations [17].Using 53BP1 and RAD51 as a marker for respectively NHEJ and HR, we demonstrate that OPM-2 and RPMI-8226 cells have a certain degree of basal activity of both repair pathways. Upon decitabine exposure, RAD51 foci formation remained at basal level in OPM-2 cells, indicating that decitabine did not stimulate signaling towards HR in OPM-2 cells. The lack of induction of RAD51 foci in OPM-2 cells was furthermore reflected by the accumulation of OPM-2 cells in G0/G1-phase after decitabine exposure. Concordantly, 53BP1 foci formation was induced by decitabine what reflects NHEJ stimulation in an attempt to repair most of the DNA damage. Nevertheless, as RAD51 positivity remained at basal level, part of the damage might be repaired by HR repair. This is further supported by the presence of approximately 20% S-phase (BrdU positive) cells. In contrast to OPM-2 cells, both 53BP1 and RAD51 foci formation was induced by decitabine in RPMI-8226 cells. Since these cells were arrested in G2/M-phase, both NHEJ and HR seemed to be stimulated and may thus compete with each other to repair DNA [12]. Given that NHEJ is more error-prone compared to HR, the balance between the two pathways may eventually determine the occurrence of (lethal) chromosome aberrations and thus cytotoxicity [17]. However, we have to note that abnormalities in different DNA repair pathways in different cell lines may determine which pathway is dominant over another and if DNA lesions are successfully repaired. For example, RPMI-8226 and to a lesser extent OPM-2 cells have impaired capacity to successfully complete NHEJ [14]. This impaired capacity may affect the accumulation of cytotoxic chromosomal aberrations.

Similar to previous studies, we observed that the combination between decitabine and the HDACi JNJ-585 had synergistic anti-MM effects which were associated with enhanced caspase, PARP-1 and MCL-1 cleavage compared to either agent alone [25-28, 49]. We also confirmed the combinatory effects of decitabine and JNJ-585 *in vivo*. At the dose used in our study, JNJ-585 did not increase gamma-H2AX foci formation on itself, while the level of gamma-H2AX positivity between decitabine and the combination was similar. This prompted us to investigate how the DNA repair response towards decitabine is influenced in combination with JNJ-585. In RPMI-8226 cells, JNJ-585 maintained RAD51 positivity at basal levels in response to decitabine while in OPM-2 cells JNJ-585 decreased RAD51 positivity below basal levels in combination with decitabine. In contrast, 53BP1 positivity was largely unchanged by JNJ-585 in response to decitabine. This indicates that NHEJ activity remained stable in the combination conditions while HR activity decreased. The effects on cell cycle progression in the combination also support this. JNJ-585 consistently induced a G0/G1-phase arrest. This arrest was stronger than for decitabine and was also sustained in the combination in OPM-2 and JJN3 cells. Importantly, at the time of the G0/G1-arrest, decitabine-induced DNA damage was already present as evidenced by the significant higher gamma-H2AX formation in the combination. Thus, because of the G0/G1-arrest induction (after decitabine-mediated DNA damage) by JNJ-585 and/or the inhibitory effects on HR, it seems that the cells' repair capacity becomes more and more restricted to NHEJ. This then may result in a faster and stronger accumulation of chromosomal aberrations compared to decitabine alone, hence explaining the enhanced cytotoxic effects of the combination. Collectively, we report that JNJ-585 negatively influenced HR-mediated DNA repair in response to decitabine by inducing a G0/G1-phase arrest, inhibiting RAD51 foci formation and/or downregulating expression of key HR genes. These effects likely shift the balance of DNA repair to NHEJ and are in favor of the hypothesis that DNA repair is a factor that determines the onset of apoptosis by decitabine. The effect of JNJ-585 on RAD51 are in line with results from others demonstrating that HDACi downregulate RAD51 in colon cancer and Chinese hamster ovary cells and inhibit irradiation-mediated RAD51 foci formation [50, 51]. In analogy, the HDACi SNDX-275 and LBH-589 have previously been shown to enhance cytotoxic effects of melphalan and doxorubicin in MM [52, 53]. Interestingly, the effects of JNJ-585 on HR genes are similar to what has been described for bortezomib, namely downregulation of HR-related genes (so called “BRCAness state) [54]. Our results imply that JNJ-585 may also be a candidate compound to interfere with HR-mediated DNA repair especially in combination with DNA damaging agents and point towards the HR pathway being a promising target to pursue for MM treatment [15].

Finally, we used the RAD51 specific inhibitor B02 to prove the role of RAD51 in decitabine-induced DDR. B02 alone was cytotoxic indicating that RAD51 is a potential target in MM, even in the absence of DNA damaging agents. Furthermore, B02 could sensitize both RPMI-8226 and OPM-2 cells to decitabine. This is in agreement with the effect of JNJ-585 and again indicates that by inhibiting RAD51, the repair balance may be shifted towards NHEJ leading to more cytotoxicity. Unexpectedly, ABT-888, sensitized RPMI-8226 but not OPM-2 cells to decitabine. ABT-888 (Veliparib) is a PARP1/2 inhibitor and is a promising agent in (pre-) clinical development for solid and hematological malignancies especially in combination with DNA damaging agents [35, 54-56]. PARP proteins are mainly implicated in single strand break repair but have been shown to be important for double strand break repair as well [57-59]. More specifically, PARP proteins promote HR by detecting collapsed replication forks and recruiting DNA repair factors important for HR [60]. In addition, both NHEJ-inhibiting and -promoting roles have been described for PARP [61, 62]. Our observation that ABT-888 enhances decitabine-mediated apoptosis suggests that PARP1/2 protects cells from decitabine-induced DNA damage, possibly by inhibiting NHEJ [58].

Currently, clinical trials with HDACi are ongoing. In MM, panobinostat in combination with dexamethasone and bortezomib was recently shown to overcome bortezomib-resistance in relapsed/refractory MM patients [19, 63]. Another HDACi, vorinostat, in combination with bortezomib had also beneficial effects in refractory/relapsed patients [18]. Furthermore, a dose escalation study with JNJ-585 (quisinostat) in combination with bortezomib and dexamethasone showed a good safety profile and clinical activity in relapsed MM patients [64]. However, no reports on clinical trials using decitabine in MM have been published, probably because of absence of significant clinical activity. This may be explained by the lack of decitabine incorporation in mature MM cells which in general have a low proliferation rate. However, proliferation is augmented in advanced stages and is considered a bad prognostic factor [65]. In addition, during normal development, plasma cells transit through an immature pre-plasmablast stage characterized by a high proliferation rate [66, 67]. Recently, a similar MM subpopulation mediating therapeutic resistance to bortezomib has been identified in MM patients [68]. Thus, when proliferation is higher (ie. in advanced stages or in pre-plasmablasts), decitabine will probably be actively incorporated what may result in a clinical response, especially in combination with a HDACi or DNA repair inhibitors. Interestingly, Caraux *et al.* reported the existence of a residual MM cell population after high dose melphalan and autologous stem cell transplantation [69]. Further characterization of this MM population is desirable as it represents cells that survived induction therapy with bortezomib and dexamethasone as well as high-dose melphalan and transplantation. These cells thus may have an immature phenotype, a high proliferation rate and ongoing DNA repair activity. Therefore, the use of other DNA damaging agents such as decitabine and agents that interfere with DNA repair such as JNJ-585 or B02 may be useful to target this population at the right moment during therapy (ie. post-transplant). Furthermore, it has been suggested that plasticity between pre-plasma cells and plasma cells is epigenetically regulated and underlines clinical resistance [70]. Better understanding of this epigenetic program will be necessary to find out whether epigenetic modulating agents may be useful to elucidate key elements of this epigenetic program or may have a favorable effect on pre-plasma cell – plasma cell transition in terms of circumventing resistance.

In conclusion, we report that DNA damage plays an important role in the mechanisms of decitabine-mediated cytotoxicity in MM. Notably, decitabine is less potent than for example melphalan in inducing DNA damage but decitabine also has epigenetic effects, definitely in combination with HDACi. Furthermore, interfering with DNA repair pathways using HDACi or more specific DNA repair inhibitors appears a promising strategy to enhance decitabine-mediated cytotoxicity. A direct implication of the data is that deeper understanding of DNA repair processes in MM cells can lead to more targeted combinations with DNA damaging agents.

## MATERIAL AND METHODS

### Cell Lines

HMCLs were obtained from ATCC (Molsheim, France). RPMI-8226, OPM-2 and JJN3 were cultured in RPMI-1640 medium (Lonza, Basel, Switzerland) with 10% FCS (Biochrom AG, Berlin, Germany) and supplements (100U/ml penicillin/streptomycin and 2mM L-glutamine (Lonza)). NCI-H929 cells were grown in RPMI-1640 medium with 20% FCS (Biochrom AG), supplements plus 1mM Na-Pyruvate (Lonza) and 55µM β-mercaptoethanol (Sigma, Bornem, Belgium). Authenticity of HMCLs was regularly confirmed by short-tandem repeat analysis.

### Mice

C57BL/KaLwRij mice were purchased from Harlan CPB (Horst, The Netherlands) and housed under conventional conditions. Mice were treated according to the conditions approved by the Ethical Committee for Animal Experiments of the Vrije Universiteit Brussel (license no. LA1230281).

### Compounds

Decitabine (Dacogen) and JNJ-26481585 (quisinostat; JNJ-585) were kindly provided by Johnson & Johnson (Beerse, Belgium). Melphalan and B02 were obtained at Sigma. ABT-888** was obtained from Selleckchem (Munich, Germany). Decitabine, JNJ-585, ABT-888 and B02 were dissolved in dimethylsulfoxide. Melphalan was dissolved in acidified ethanol. For *in vivo* experiments, decitabine and JNJ-585 were used as a filter sterilized 10% hydroxypropyl-cyclodextran suspension.

### Apoptosis assay

Apoptosis was measured by flow cytometry (FACSCanto, BD Biosciences, Franklin Lakes, USA) using AnnexinV-FITC and 7'-aminoactinomycin D (7'AAD) (BD Biosciences) according to manufacturer's instructions. Results were analyzed using FACSDiva software (BD Biosciences).

### BrdU incorporation and cell cycle analysis

Bromo-deoxyuridine (BrdU; 1mg/ml) (Roche Diagnostics, Vilvoorde, Belgium) was administered 2 hours prior to harvest. Next, cells were washed and fixed in paraformaldehyde overnight. The following day, cells were incubated with HCl and washed before anti-BrdU-FITC antibody (Roche diagnostics) was added. Next, cells were washed and incubated with a propidium iodide (PI) solution containing 0.1% Triton-X100 (Merck, Barmstadt, Germany), 1mg/ml sodium nitrate (Merck), 100µg/ml RNaseA (Boehringer Ingelheim, Germany) and 50µg/ml PI (Sigma). Cells were analyzed by flow cytometry (FACSCanto) using FACSDiva software.

### Western blot

Western blot was performed as described previously [29]. The antibodies used were all purchased from Cell Signaling (Bioké, Leiden, The Netherlands). The following clones of antibodies were used: caspase-3 (#9665), caspase-9 (#9502), caspase-8 (#9746), BIM (C34C5, #2933), MCL-1 (D35A5, #5453), p27 (D37H1, #3688), SKP-2 (L70, #4313) and ACTIN (#4967).

### Quantitative real-time PCR

After indicated time-points, cells were harvested and RNA was extracted by the RNeasy mini kit (Qiagen, Venlo, The Netherlands). 1µg of total RNA was converted into cDNA using the First-strand cDNA synthesis kit (VWR International, Leuven, Belgium). Quantitative real-time PCR was performed and analyzed as previously described [29]. Primers for BRCA1, BRCA2 and RAD51 were purchased at Thermo Scientific (Aalst, Belgium) and ABL-1 primers were bought at Integrated DNA Technologies (Leuven, Belgium). Primer sequences were as follows (5'-3'): BRCA1: forward: ACCGTTGCTACCGAGTGTCTG; reverse: GTGATGTTCCTGAGATGCCTTTGC; BRCA2: forward: AACCGTGTGGAAGTTGCGTA TTG; reverse: GGCTCCCGTGGCTGGTAAATC; RAD51: forward: TCAAGTGGATG GAGCAGCGATG; reverse: TGGCAGTCACAACAGGAAGAGG. ABL1: forward: TTGTGGCCAGTGGAGATAAC; reverse: GTTTGGGCTTCACACCATTC.

### Immunofluorescence staining and microscopy

Cells were plated at a density of 20.000/ml three days before treatment. After indicated timepoints, cytospins were made and stored at -20°C. The staining protocol was as follows: cytospins were fixed in 4% paraformaldehyde and washed with Tris-NaCl 0.05% Triton-X100 (Merck). Next, cytospins were blocked by 2% donkey serum and incubated overnight with the primary antibodies; gamma-H2AX (20E3, #9718; Bioké), RAD51 (ABE257; Millipore, Overijse, Belgium), 53BP1 (100-304; Novus Biologicals, Cambridge, UK). Next, cytospins were washed and incubated for 1 hour with donkey anti-rabbit-FITC (Jackson ImmunoResearch, St-Martens-Latem, Belgium). After washing, cytospins were mounted with Vectashield (Thermo Scientific) containing 4,6-diamidino-2-phenylindole (DAPI). Immunofluorescence was observed using a Nikon Eclipse 90i with a 60X objective magnification. Pictures were taken using a Nikon DS-Ri1 and analyzed using the ImageJ macro PZ-FociEZ. Briefly, DAPI was used to define the nuclei. Next, foci were counted in the nuclei by analyzing local maxima in fluorescence intensity. At least 100 nuclei were analyzed and nuclei with at least 10 foci were scored as positive.

### Prophylactic treatment of 5T33MM mice

The 5T33MM model was maintained as previously described [30]. At day 0, naive C57BL/KaLwRij mice were intravenously injected with 5×10^5^ 5T33MM cells. Mice were treated with decitabine starting at day 1 (intraperitoneal injection, 6 days/week). For the combination experiment, JNJ-585 was administered subcutaneously at 1.5mg/kg once every other day. Mice were sacrified after 3 weeks, when vehicle treated 5T33MM mice showed clear signs of morbidity (hind limb paralysis). For the survival study, the treatment schedule continued until each individual animal showed signs of morbidity. Blood was collected for serum M-spike determination and BM was isolated from hind legs to quantify tumor burden as previously described [31].

### Graphical analysis and statistics

Graphical and statistical analyses were done using Sigmaplot 11.0 (Systat software Inc., San Jose, USA). Student t-test or Mann-Whitney test was used to compare parametric or non-parametric data, respectively. Survival curves were created by Kaplan-Meier analysis. Survival probabilities were compared by a log-rank test. P values of < 0.05 were considered statistically significant.

## SUPPLEMENTARY FIGURES


