# Supplementary material for: The role of DNA damage and repair in decitabine-mediated apoptosis in multiple myeloma
Source: Oncotarget. 2014 Mar 22;5(10):3115–29. doi: 10.18632/oncotarget.1821 (PMC4102796; doi:10.18632/oncotarget.1821)
Supplement: Supplementary file 1 [file oncotarget-05-3115-s001.pdf]

The Role of DNA Damage and Repair in Decitabine-Mediated Apoptosis in Multiple Myeloma – Maes et al

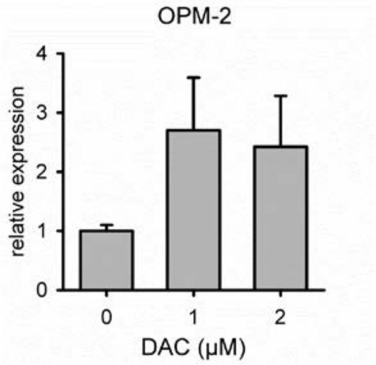

Supplementary Figure S1: DAC induced BIM mRNA expression in OPM-2 cells. OPM-2 cells were treated with DAC for 3 days. BIM mRNA expression was determined by quantitative real-time expression. Primers for BIM: (5'-3') forward: GCCACTACCACCACTTGATTCTTG; reverse: CCGTGATTGCCTTCAGGATTACC. Data are presented relative to untreated cells. Bars and error bars represent mean and SD from 3 experiments. * indicates p<0.05.

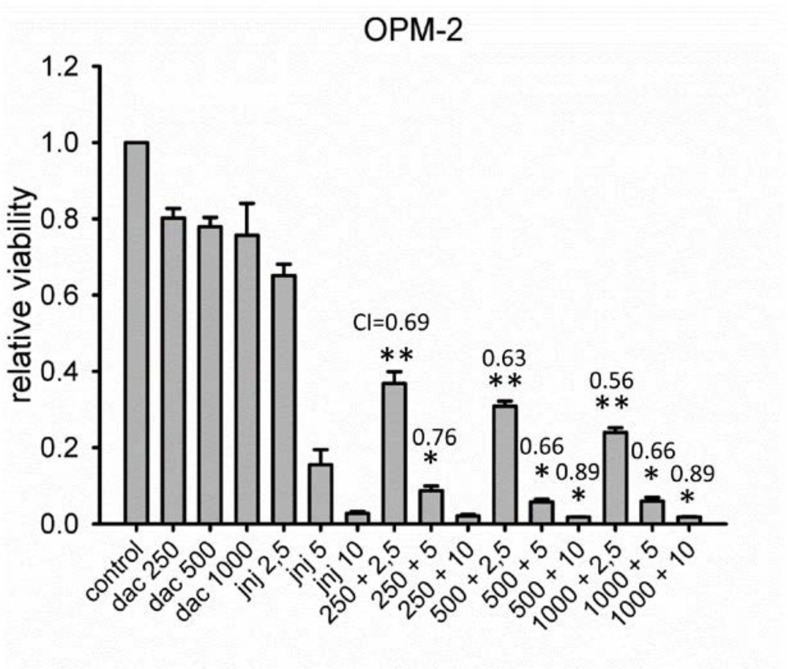

Supplementary Figure S2: DAC and JNJ-585 have synergistic anti-myeloma effects. OPM-2 cells were treated with three doses of DAC (250, 500, 1000nM) or JNJ-585 (2.5, 5, 10nM) and the combination. Effects on viability were determined by Cell-Titer Glo assay according to manufacturer's instructions. Briefly, cells were seeded in 96-well plates ($3x10^5$ cells) and treated for three days. Next, the substrate was added and the bioluminescent signal was quantified with the GloMax 96 plate luminometer (Promega). Experimental conditions were performed in triplicate. Results are expressed as the relative viability compared to untreated cells. Bars and error bars are mean and SD of three independent experiments. Synergy and combination indexes (CI) were determined using Calcusyn software. CI > 1.0 indicates an antagonistic effect, CI= 1.0 an additive effect and CI < 1.0 a synergistic effect. * means p<0.05, ** means p<0.01.

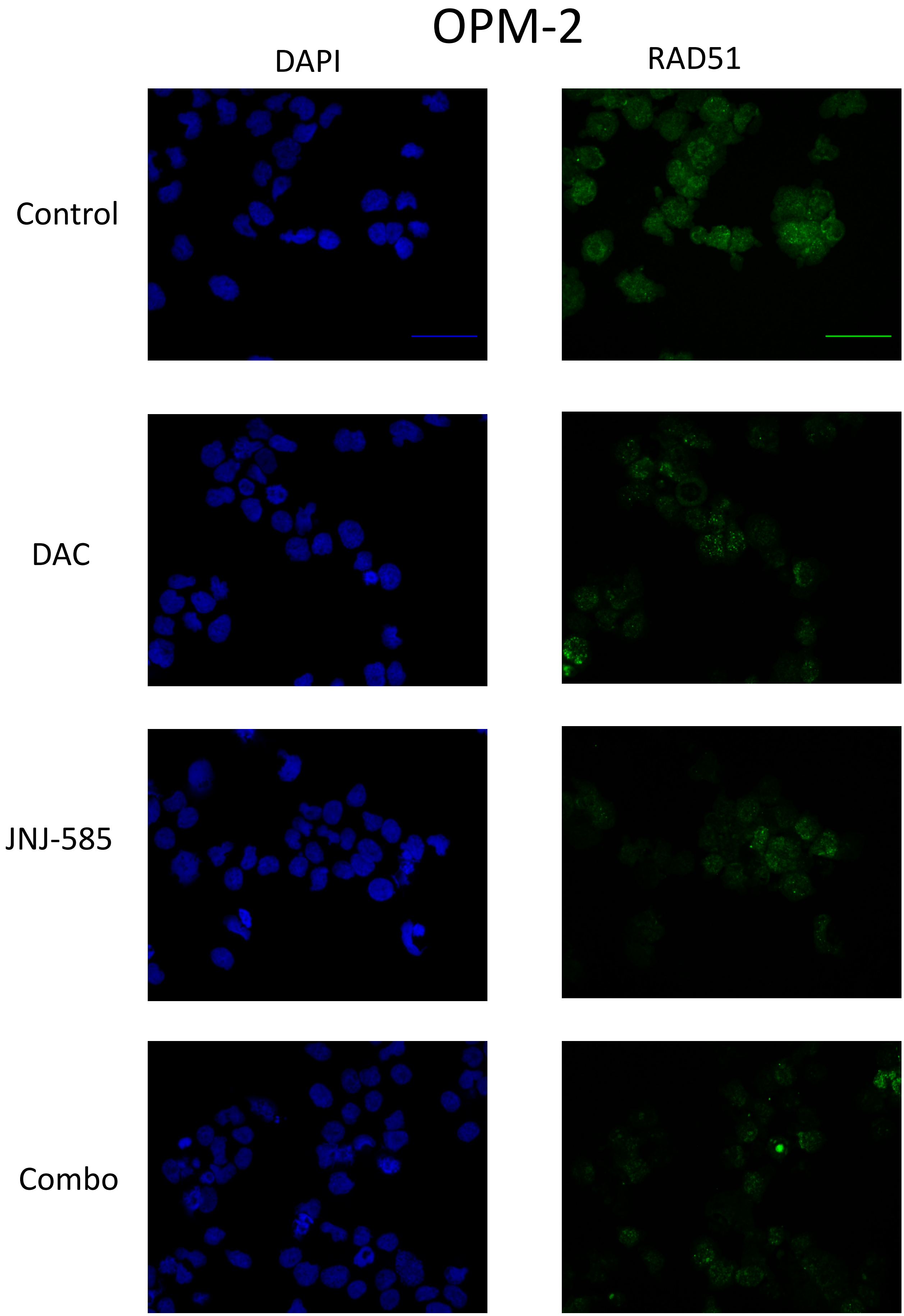
OPM-2
DAPI
RAD51
Control
DAC
JNJ-585
Combo

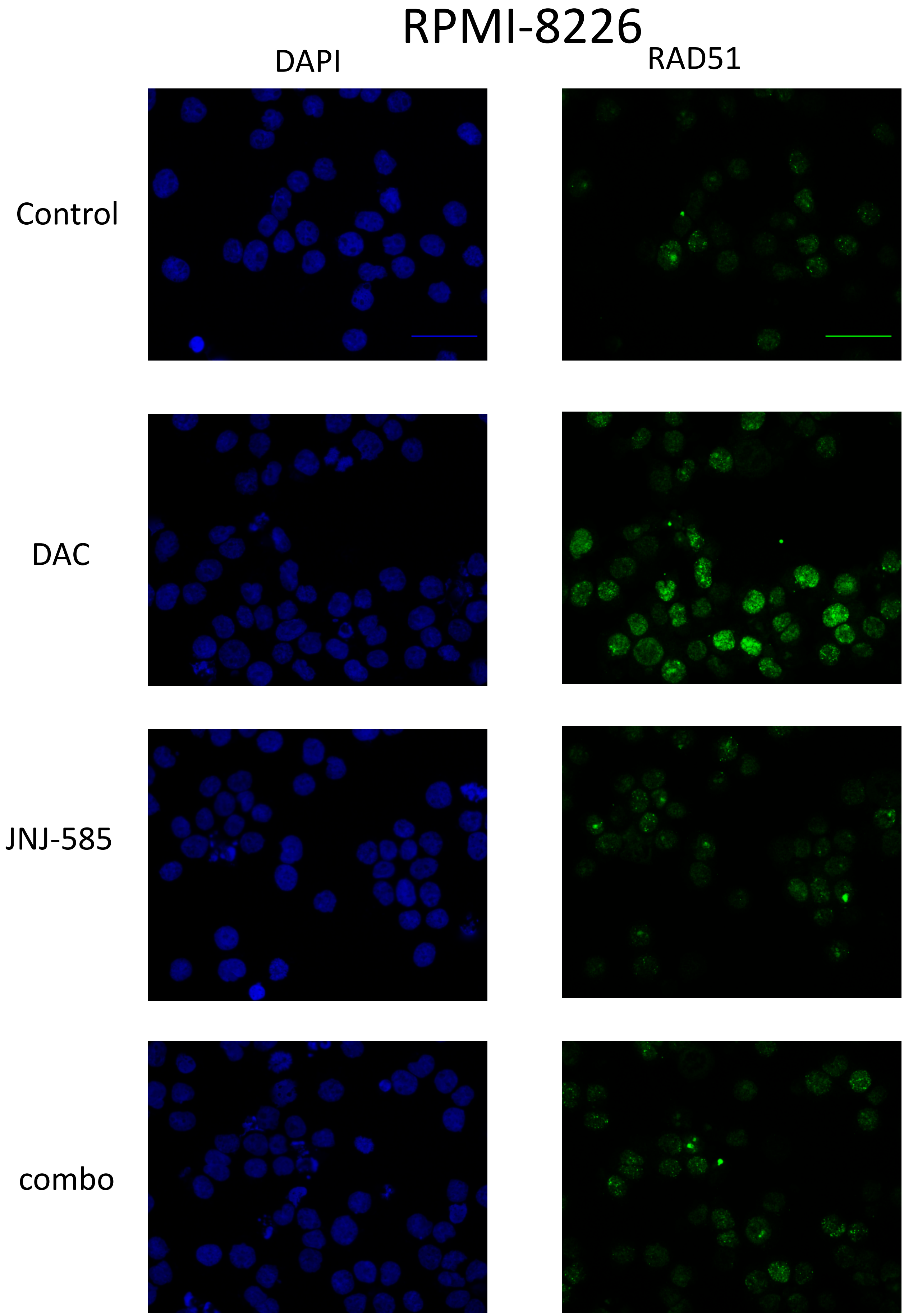
RPMI-8226
DAPI
RAD51
Control
DAC
JNJ-585
combo

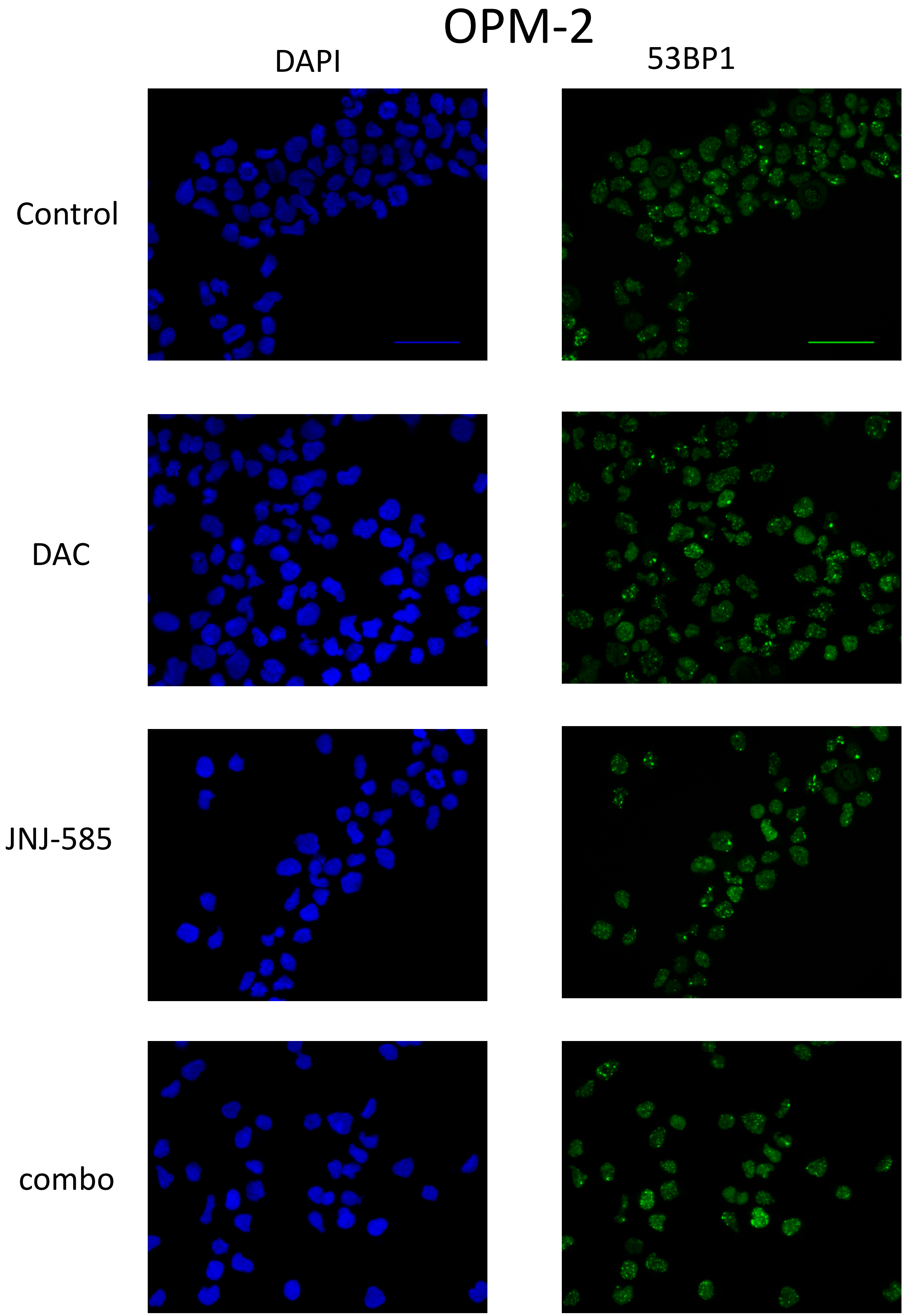

OPM-2
DAPI
53BP1
Control
DAC
JNJ-585
combo

RPMI-8226

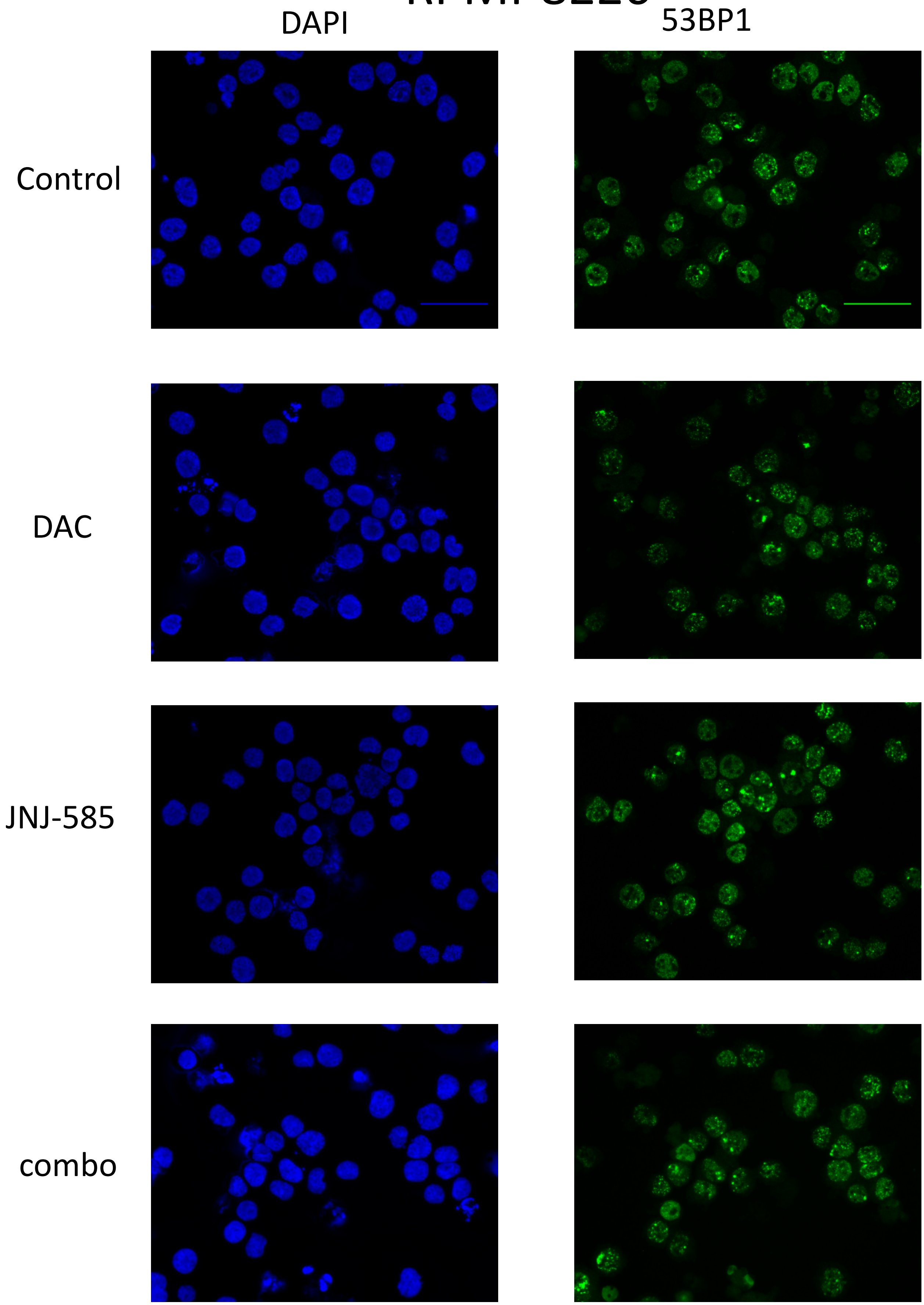

Supplementary Figure S3-S6: JNJ-585 influenced DAC-mediated DNA repair responses. Cells were treated with DAC and/or JNJ-585 for 1 day. Doses for OPM-2 were 1µM DAC and 2.5nM JNJ-585; for RPMI-8226 1µM DAC and 5nM JNJ-585. Next, cytospins were made and stained for DAPI and RAD51 (S3, S4) or 53BP1 (S5, S6). Immunofluorescent pictures representative of one experiment are shown. Scale bar = 50µm.
